# Alteration of Cytoskeleton Morphology and Gene Expression in Human Breast Cancer Cells under Simulated Microgravity

**DOI:** 10.22074/cellj.2020.6537

**Published:** 2019-09-08

**Authors:** Florian Strube, Manfred Infanger, Markus Wehland, Xenia Delvinioti, Alexander Romswinkel, Carlo Dietz, Armin Kraus

**Affiliations:** Department of Plastic, Aesthetic and Hand Surgery, Otto-von-Guericke-University, Magdeburg, Germany

**Keywords:** Breast Neoplasms, Cytoskeleton, Proto-Oncogenes, Tumor Suppressor Genes, Weightlessness Simulation

## Abstract

**Objective:**

Weightlessness simulation due to the simulated microgravity has been shown to considerably affect behavior of tumor cells. It is aim of this study to evaluate characteristics of human breast cancer cells in this scaffold- free 3D culture model.

**Materials and Methods:**

In this experimental study, the cells were exposed to simulated microgravity in a random- positioning machine (RPM) for five days. Morphology was observed under phase-contrast and confocal microscopy. Cytofilament staining was performed and changes in expression level of cytofilament genes, proliferation/differentiation genes, oncogenes and tumor suppressor genes were detected by quantitative reverse transcription polymerase chain reaction (qRT-PCR), followed by western blot confirmation.

**Results:**

After five days, distinct spheroid formation was observed. Rearrangement of the cytoskeleton into spherical shape was visible. *VIM* gene expression was significantly up-regulated for adherent cells and spheroids (3.3x and 3.6x respectively, P<0.05 each). *RHOA* also showed significant gene up-regulation for adherent cells and spheroids (3.2x and 3.9x respectively, P<0.05 each). *BRCA* showed significant gene up-regulation in adherent cells and spheroids (2.1x and 4.1x respectively, P<0.05 each). *ERBB2* showed significant gene up-regulation (2.4x, P<0.05) in the spheroids, but not in the adherent cells. *RAB27A* showed no significant alteration in gene expression. *MAPK*) showed significant gene up-regulation in adherent cells and spheroids (3.2x, 3.0x, P<0.05 each). *VEGF* gene expression was down-regulated under simulated microgravity, without significance. Alterations of gene expressions could be confirmed on protein level for vimentin and MAPK1. Protein production was not increased for BRCA1, human epidermal growth factor receptor 2 (HER2) and VEGF. Contradictory changes were determined for *RHOA* and its related protein.

**Conclusion:**

Microgravity provides an easy-to handle, scaffold-free 3D-culture model for human breast cancer cells. There were considerable changes in morphology, cytoskeleton shape and gene expressions. Identification of the underlying mechanisms could provide new therapeutic options.

## Introduction

Breast neoplasms are still a major cause of morbidity and mortality in the western world. To identify new mechanisms that could provide therapeutic targets, experiments in 2-dimensional (2D) cell culture struggle with various limitations. Phenotype alteration and loss in 2D cultures has been described for several cell types. It has been described for somatic cells such as tenocytes (1) and mesenchymal stem cells (2). Space flights have shown to impose significant alterations on human and animal organisms, as well as cell cultures. On the organism, various effects such as loss of bone mineralization or changes in blood pressure regulation are well known. 

In different cell types, loss of attachment from the otherwise indispensable culture surface and transition to a state of 3-dimensional (3D) formations, so-called “spheroids“, has been reported. This was observed during flights beyond theearth atmosphere (3), during parabolic flight maneuvers (4), in addition to the simulated microgravity on earth. Hereby, 2D rotating clinostats or 3D rotating random positioningmachines (RPM) are in use. In both, simulated microgravity on earth and during space flights, several effects of microgravity on breast cancer cells have been reported, especially with regards to invasion, adhesion and metastasis formation (5). 

Microgravity has also been reported to induce mitochondrial activity in breast cancer cells as a reaction to oxidative stress (6). Further studies by our group have shown alterations particularly with regard to the cytoskeleton arrangement (7). Microgravity therefore seems to be an attractive 3D cell culture model to study migration and invasiveness in breast cancer. We performed experiments where we exposed breast cancer cells to simulated microgravity. We studied cytoskeleton morphology as well as gene and protein expression levels related to tumor differentiation, proliferation and invasion. In this study, we aimed to provide new insights into these mechanisms in order to identify potential targets for new therapeutic strategies. 

## Materials and Methods

The present work was designed as an experimental laboratory study (level of evidence V). 

### Cell culture

Human breast cancer cells (adenocarcinoma, CRL2351) were obtained from ATCC^©^ (Wesel, Germany). All experiments were performed on this commercially available cell line, so no Ethical Committee approval was necessary. The cell line is negative estrogen receptor and it overexpresses the HER2/neu oncogene. The cells were firstly expanded under 2D-conditions in T125 flasks (Sarstedt, USA). Ham´s F12-media (Gibco, Germany) supplemented with 5% fetal calf serum (FCS, Biochrom AG, Germany) and 1% penicillin/streptomycin (Biochrom, Germany) was used. The medium was changed three times per week. For this experiment, 1×10^6^ cells were counted by hemocytometer and added to six T125 flasks, as the experimental group in the RPM, and to the same number of flasks for the control group under 1g conditions. For cytoskeleton staining, the cells were seeded with a density of 1×10^5^ per cm^2^ to slide flasks (Thermo Scientific, Germany). 

### Random positioning machine

Weitghtlessness simulation was generated using an RPM. The RPM (developed by University of Applied Sciences, Northwestern Switzerland) was run with a commercially available incubator at 37°C and 5% CO_2_. The device was operated in a random walk modus using an angular velocity of 60°/seconds. The method was intensively investigated and published earlier (8). Six flasks of T125 cm^2^ were attached as much as possible to the center of RPM machine, and the samples were rotated for the selected time period (five days). Static, non- rotated controls were exposed to the same environmental conditions nearby the device. The RPM machine was rebooted once per 24 hours to ensure proper operation. Interruption was kept as short as possible every time. 

### Phase contrast microscopy 

Phase contrast microscopy was performed for visual observation of viability and morphology of the cells, and for detection of potential spheroids. A Leica microscope (Leica Microsystems GmbH, Germany) was used. Pictures were taken with a Canon EOS 60D (Canon GmbH, Germany). 

### Cytoskeleton staining

In terms of cytoskeleton analysis, the cells exposed to simulated microgravity in the RPM for five days were investigated in the slide flasks. Filamentous actin (F-actin) was analyzed by visualization of phalloidin-stained cells (PromoKine, USA). Both adherent cells and spheroids were fixed with 4% paraformaldehyde for 10 minutes and permeabilized with 1% Triton-X for 5 minutes. Nonspecific binding was blocked by incubation with 1% bovine serum albumin (BSA). Staining was performed by incubation of the slides with 6.6 µM solution of a phalloidin/Alexa Fluor 488 conjugate (Thermo Fisher Scientific, Germany) for 30 minutes at room temperature, followed by thorough washing with phosphate buffere saline (PBS) solution. Nuclei were counterstained with 4',6-diamidine-2-phenylindol (DAPI, Thermo Fisher Scientific, Germany) at 0.1 µg/ml concentration for 1 minute. The samples were mounted with Vectashield mounting medium (Vector Laboratories, USA).

### Confocal microscopy 

Confocal microscopy of the slides stained for F-actin was performed with a Zeiss 510 META inverted confocal laser scanning microscope (Zeiss, Germany). Excitation and emission wavelengths were 485 nm/560 nm, respectively.

### RNA and protein isolations, quantitative reverse transcription polymerase chain reaction and western blot 

#### RNA isolation 

An aliquot of cells was frozen in liquid nitrogen for subsequent lysis and protein isolation as described further below. RNA isolation and quantitative reverse transcription polymerase chain reaction (qRT-PCR) were done according to standard protocols following the manufacturer’s manual. RNA isolation was performed with the AllPrep DNA/RNA/ Protein Mini^©^ Kit (Qiagen, Germany) according to the manual. The cells were brought into suspension from the culture plate surfaces by adding 0.025% trypsin (Sigma- Aldrich, Germany). The cell suspension was spun down in an RNase-free tube for five minutes at 300 g. Next, 350 µl of the lysis buffer RLT was added to the pellet to induce cell lysis. Remaining RNases were inactivated by 1% ß-mercaptoethanol addition. The lysate was vortexed for 1 minute to obtain a homogenous lysate. Thereafter, it was stabilized by adding 100% ethanol in an equal volume to the lysate. The liquid was then transferred to an RNeasy Spin Column and centrifuged in a micro-centrifuge at 10,000 rpm for 15 seconds. The flow-through was put aside for further protein isolation as described below. Afterwards, 700 µl of the buffer RW1 (washing buffer) was given to the spin column, followed by centrifugation at 10,000 rpm for 15 seconds. The flow-through was discarded again. The washing buffer RPE^©^ was added to the spin column at a volume of 500 µl and the column was centrifuged at 10,000 rpm for 15 seconds. This step was repeated after discarding the flow-through. The RNA was then eluted into an RNase-free collection tube by centrifugation with 30 µl of RNase-free water. The RNA was quantified photometrically by measuring the absorbance at 260 nm with a SpectraMax M2 device (Molecular Devices, USA). 

### Reverse transcription

Reverse transcription was done using the First Strand cDNA Synthesis Kit (Thermo Scientific, USA) according to the manual. Briefly, for each sample, 3 µg total RNA, random hexamer primers and nuclease-free water were mixed together to an overall volume of 11 µl. To this volume, reaction buffer, RNase inhibitor, oligonucleotides and reverse transcriptase were added to a total volume of 20 µl. All steps were performed under refrigeration on ice. The mix was kept for five minutes at 25°C, followed by 60 minutes at 42°C. The reaction was stopped by incubation at 70°C for five minutes. The complementary DNA (cDNA) was stored at -20°C for less than one week before performing further experiments. 

### Quantitative reverse transcription polymerase chain reaction 

qRT-PCR was utilized to determine relative expression of the target genes, such as proto-oncogenes and tumor suppressor genes, as shown in Table 1. The SYBR® Green PCR Master Mix (Applied Biosystems, Germany) and the 7500 Real-Time PCR System (Applied Biosystems, Germany) were used. 10 µl master mix, 1 µl of each forward and reverse primers at the concentration of 400 nM and 1-8 µl cDNA and RNase free water, in relation to the input-amount of RNA, were mixed together. Cycling steps were executed as follows after activation of uracil-DNA glycosylase (50°C for two minutes) and DNA polymerase (95°C for 2 minutes): 95°C for 15 seconds and 60°C for 1 minute (40 cycles). Absence of primer dimers was confirmed by checking dissociation curves. cDNA-selective primers were collected from Harvard primer database (https://pga.mgh.harvard.edu/ primerbank) and were supplied by TIB Molbiol (Germany). All samples were measured as triplicates. 18S rRNA was used as housekeeping gene. The comparative C_T_ (ΔΔC_T_) method was used to calculate relative transcription levels of the target genes. The control group was defined as 100%. Primer sequences are as shown in Table 1. The experiments were performed in five replicates. 

**Table 1:** Quantitative reverse transcription polymerase chain reaction(qRT-PCR) primer sequences. All primers were obtained from Harvardprimer bank (https://pga.mgh.harvard.edu/primerbank/)


Gene	Primer sequence (5ˊ-3ˊ)

*VIM*	F: GACGCCATCAACACCGAGTT
	R: CTTTGTCGTTGGTTAGCTGGT
*RHOA*	F: CTCGCTCAGTGCGAAGACAA
	R: CATTCTCTGACGACATTTTCCCT
*BRCA1*	F: GCTCGTGGAAGATTTCGGTGT
	R: TCATCAATCACGGACGTATCATC
*ERBB2*	F: CCTCTGACGTCC ATCGTCTC
	R: CGGATCTTCTGCTGC CGTCG
*RAB27A*	F: GCTTTGGGAGACTCTGGTGTA
	R: TCAATGCCCACTGTTGTGATAAA
*MAPK1*	F: TACACCAACCTCTCGTACATCG
	R: CATGTCTGAAGCGCAGTAAGATT
*VEGF*	F: AGGGCAGAATCATCACGAAGT
	R: AGGGTCTCGATTGGATGGCA
*18S rRNA*	F: ATGGCGGCGTCTGTATTAAAC
	R: AGAACCATATCGCTCCTGGTAT


### Western blotting

Protein and RNA isolations were simultaneously performed using the AllPrep DNA/RNA/Protein Mini^©^ Kit, as described above. The flow-through preserved from the respective elution step, described above, was mixed with an equal volume of the buffer APP^©^ from the extraction kit and kept for 10 minutes at room temperature after meticulous mixing for protein precipitation. The suspension was centrifuged for 10 minutes at maximum speed and the supernatant was carefully pipetted off to obtain a protein pellet. The pellet was dried at room temperature for 10 minutes. The pellet was next dissolved again in 100 µl of the buffer ALO^©^ and heated to 95°C for this purpose. The solution was centrifuged again at full speed for one minute to remove remaining precipitates and debris. The protein solution was frozen at -20°C until further use. Gel electrophoresis, transblotting and densitometry were performed according to the standard protocols. Isolated protein was incubated for 10 minutes with sodium dodecyl sulfate (SDS)-gel loading buffer (consisting of 1 M Tris base, pH=6.8, 1% clycerol, 10% SDS, 0.1% bromophenol blue freshly added to 0.05% ß-mercaptoethanol and 1% protease inhibitors, all purchased from Roche, Germany). The sampleswere denatured at 95°C for five minutes. Afterwards, the probes were loaded together with the prestained page rule (Thermo Scientific, USA) onto a 10% SDSpolyacrylamide gel followed by electrophoresis and semi-dry blotting onto 0.45 µm nitrocellulose membranes (Whatman, Germany). Primary antibodies were used in blocking reagent with following dilutions: rabbit polyclonal anti-vimentin (1:2000), rabbit polyclonal antiRhoA(1:500), rabbit polyclonal anti-Her2 (1:1000), rabbit polyclonal anti-RAB27A (1:1000), rabbit polyclonal antiMAPK1 (1:1000), all obtained from Origene, USA, as well as rabbit polyclonal anti-BRCA1 (1:10000, Milipore, USA), rabbit polyclonal anti VEGF-A (1:1000, Thermo Fisher, USA). All antibodies were certified for western blot reactivity in human specimens by the suppliers. 

The secondary antibody was included in the “BM Chemiluminescence Western Blotting Kit mouse/rabbit” (Roche, Germany). The blots were stripped at 50°C for 30 minutes with stripping buffer (Restore Western blot stripping buffer, Thermo scientific, USA), washed and re-incubated with anti-GAPDH antibody (1:1000, Cell Signaling, Germany). Blots were analyzed by the Alpha- Ease® FC Imaging System (Alpha Innotech, Germany). The experiments were performed in three replicates. 

### Statistical analysis

All statistical analyses were done using SPSS 21.0 (SPSS, Inc., USA, 2012). The groups were tested with the Mann- Whitney U test. The data are shown as means ± standard deviation (SD). A P<0.05 was considered significant. 

## Results

A simulated average gravity value as low as 0.003 g was calculated by the RPM software.

### Light microscopy

**Fig.1:**
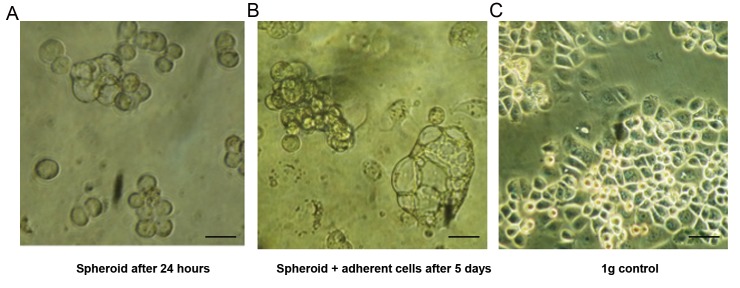
Analysis of spheroid formation of breast cancer cells under simulated microgravity (0.003 g) after one and five days, under light microscopy. A. Formation of small spheroids can be observed after 24 hours, B. After 5 days, size of the cluster to tubular shaped spheroids increased, while part of the cells remained attached to the culture flask surface, and C. In the control group under 1 g conditions, the cells showed typical shape with flat morphology and rectangular to hexagonal borders (scale bar: 50 µm).

After 24 hours, formation of smaller spheroids, consisting of approximately 10-20 cells, was observed (Fig .1A). After five days, a considerable number of cells had detached from the culture surface, forming cluster shaped spheroids consisting of 30-50 cells (Fig .1B). Breast cancer cells showed rounded morphology with no obvious signs of impaired viability, according to preliminary trypan blue staining. This shows good cell viability without significant difference between the cells at 1g and those under simulated microgravity (data not shown). In the control group, under 1g conditions, the cells showed typical flat morphology with rectangular to hexagonal borders, attached to the culture surface (Fig .1C). 

### Confocal microscopy

**Fig.2:**
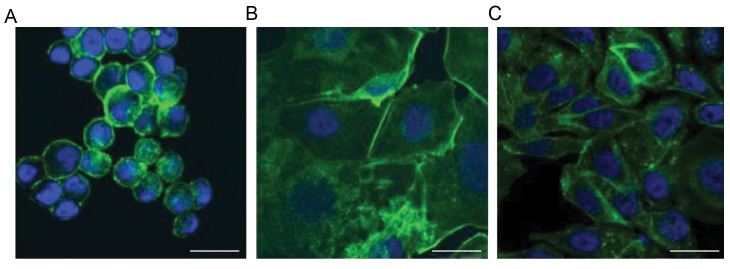
Actin staining of breast cancer cells. **A.** In the spheroids under simulated microgravity, actin filaments arranged in a spherical shape with accentuation in the area of the cell membrane, **B.** In the adherent cells under simulated microgravity, spherical orientation beginning of the actin filaments was observed, and **C.** In the 1 g control group, actin filaments were arranged in a longitudinal manner with uniform distribution among the cytoplasm (scale bar: 25 µm).

After five days, the breast cancer cells showed under microgravity spherical rearrangement of actin filaments with accentuation of the filaments in the region of cell membrane (Fig .2A). In the adherent cells under simulated microgravity, a tendency towards spherical orientation of the actin filaments could be observed, while that is less pronounced than spheroids (Fig .2B). Under the condition of 1 g, actin filaments were arranged in a longitudinal manner with more uniform distribution of the filaments inside the cytoplasm (Fig .2C). 

### Quantitative reverse transcription polymerase chain reaction

**Fig.3:**
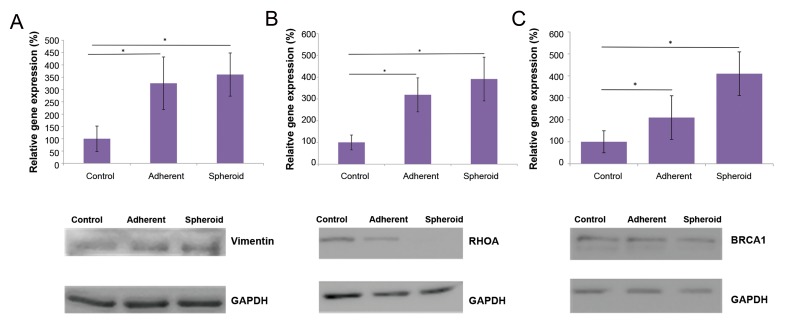
Alteration in gene expression and protein production under simulated microgravity. **A.** *VIM* as a component of the cytoskeleton showed significantly up-regulation in the both attached cells and spheroids, after five days (3.3x and 3.6x respectively, *P<0.05 each). *VIM* up-regulation was accompanied by increased vimentin protein production, **B.** *RHOA* also showed significant up-regulation in the both adherent cells and spheroids, under simulated microgravity (3.2x and 3.9x respectively, *P<0.05 each). In contrast, RhoA protein content was not increased under simulated microgravity, and **C.** *BRCA1* showed significant up-regulation in the both adherent cells and spheroids (2.1x and 4.1x respectively, *P<0.05 each). BRCA1 protein content was not significantly increased, as shown by western blot.

**Fig.4:**
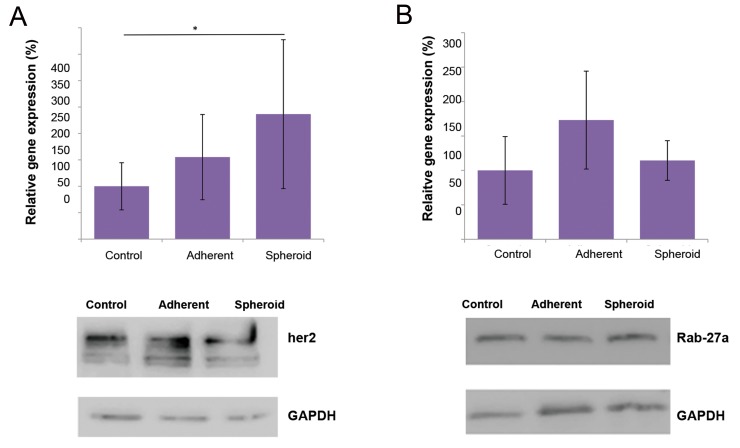
Alteration in gene expression and protein production under simulated microgravity. **A.** *ERBB2* showed significant gene up-regulation (2.4x, *P<0.05) in the spheroids, compared to the 1 g control group, while the corresponding protein production was not increased under microgravity and **B.** *RAB27A* showed no significant change in gene expression of the both adherent cells simulated under microgravity and spheroids. There was no change in Rab-27a protein content.

**Fig.5:**
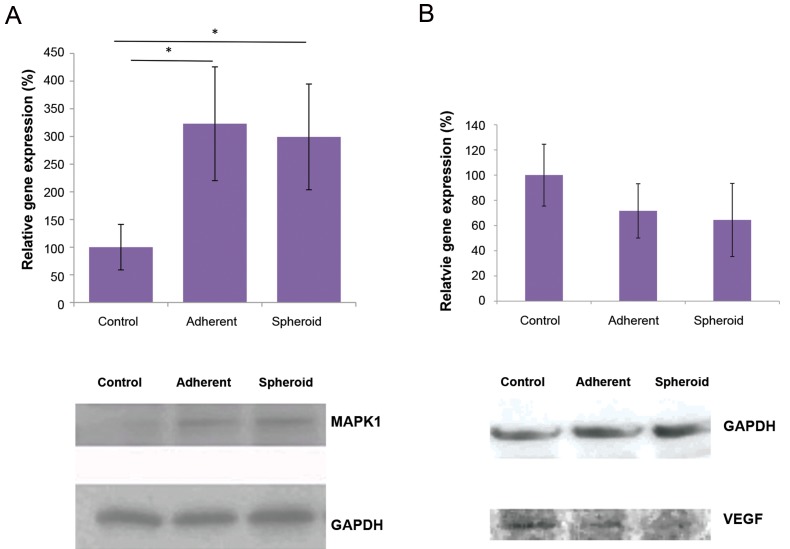
Alteration in gene expression and protein production under simulated microgravity. **A.** After five days, *MAPK1* showed significant up-regulation in the both adherent cells and spheroids under simulated microgravity (3.2x and 3.0 respectively, *P<0.05 each). Gene up-regulation was confirmed on protein level by western blot and **B.** *VEGF* was down-regulated under simulated microgravity in both gene expression and protein levels, but the values were not statistically significant.

After five days, *VIM* as a component of the cytoskeleton showed significantly up-regulated gene expression in the both attached cells and spheroids (3.3x and 3.6x respectively, P<0.05 each, Fig .3A). Correspondingly, *RHOA*, as a marker of cytoskeleton differentiation, also showed significant up-regulation in the both attached cells and spheroids under simulated microgravity (3.2x and 3.9x respectively, P<0.05 each, Fig .3B). *BRCA1* gene showed significant up-regulation in the both adherent cells and spheroids, compared to the control with 1 g (2.1x and 4.1x respectively, P<0.05, Fig .3C). *ERBB2* showed no significant up-regulation in the adherent cells under microgravity, while it was significantly up-regulated in the spheroids (2.4x, P<0.05, Fig .4A). *RAB27A*, as a KRAS-related control gene, showed no significant up-regulation in the both attached cells and spheroids under microgravity (Fig .4B). *MAPK1*, as a marker of proliferation and differentiation, showed significant up-regulation in the both adherent cells and spheroids under microgravity (3.2x and 3.0x respectively, P<0.05 each, Fig .5A). *VEGF* showed down-regulation in the both adherent cells (under simulated microgravity) and spheroids (0.67x and 0.60x respectively, Fig .5B), while it was not significant different (P=0.056 each). 

### Western blots

In line with *VIM* gene overexpression, analysis of western blot showed up-regulation of vimentin protein (Fig .3A). In the case of *RHOA*, protein expression level was attenuated under simulated microgravity gene (Fig .3B). Although *BRCA1* gene expression was significantly up- regulated in the adherent cells and spheroids, western blot analyses showed approximately similar protein levels for adherent cells, spheroids and the control (Fig .3C). *ERBB2* gene expression was significantly up-regulated in the spheroids, while the respective protein bands showed approximately equal intensities for all groups (Fig .4A). Rab-27a showed no changes in protein levels of the three groups, similar to the related gene expressions (Fig .4B). MAPK1 showed increased protein levels in the two simulated microgravity groups, which was consistent to the respective up-regulated gene expressions (Fig .5A). VEGF protein was decreased in both groups under simulated microgravity, compared to the control group (Fig .5B). 

## Discussion

Breast neoplasms are common malignancies. Thus, in terms of developing new treatments, researches on the cell culture level could be a valuable tool. It is well known that the cells tend to change their morphology, behavior and phenotype after being released from their *in vivo* environment and put into a 2D culture condition. By applying simulated microgravity, we could observe considerable changes in morphology, cytoskeleton arrangement, gene expression and protein synthesis compared to the 2D environment. For this study, five days investigation was selected, due to the following reason: preliminary experiments had shown that CRL2351 cell line was relatively fast-growing and needed medium renewal after five days, according to the both vendor´s recommendations and our own observations. Furthermore, the cells showed almost complete confluence and the necessity of passaging after five days. We aimed to avoid medium change, because this maneuver would interrupt simulated microgravity and disturb its effects to particular degree. Therefore, five days was the longest period that this cell line could continuously be exposed to interruption-free simulated microgravity. 

Compared to the several other studies utilizing 2D rotating clinostats, we used an RPM in this experiment, as a device simulating microgravity by 3D movements in space. Experiments, comparing the effects of simulated microgravity on an RPM with the space outside of earth atmosphere on cell cultures, have shown very good correlation between these two systems (9). Furthermore, we examined a cell line with unique characteristics which has not been exposed to microgravity so far, to our knowledge. In comparison with the MCF-7 cells, used in most of the studies dealing with breast cancer cells under simulated microgravity (5, 10, 11), the evaluated biologic features of CRL-2351 cell line in this study are controversial from many aspects: CRL-2351 cells are negative estrogen receptor, so the pathways usually associated with estrogen receptor signaling, like MAP kinase, will be influenced in a unique, hitherto unknown manner. Furthermore, HER2/neu is overexpressed in CRL-2351 cells, in contrast to MCF-7 cells, particularly giving us the option to evaluate the effects of simulated microgravity on this important prognostic factor. Using light microscope, observations showed 3D constitution, round to tubular-shaped formations -known as spheroids- and reported already from various tumor and somatic cell types, such as from tenocytes (12), chondrocytes (13), or thyroid cancer cells (14) .

Another group of the cells under simulated microgravity remained adherent to the culture surface, but they showed various changes in morphology, gene expression and protein production. These cells could either represent a transitional state before spheroid formation, or remain in this adherent state for reasons still needing to be elucidated. In the MDA-MB-231 breast cancer cell line, Masiello et al. (15) could observe spheroid formation after 24 and 72 hours. In other studies, significant deceleration of breast cancer cell proliferation, while they are accumulated in the G2 phase, was reported under simulated microgravity (11). In the cytoskeleton, we observed a change from the longitudinal shape of actin filaments towards spherical distribution, accentuated in the region of cell membrane. This is in accordance with the results of Kopp *et al*. who reported similar findings for another line of breast cancer cells under simulated microgravity (10), and consistent to the results of Masiello et al. (15) cited above. To explain this phenomenon, a "gravity sensor" has been proposed inherited in the cytoskeleton, which is responsive to external forces (16). Particularly for the tumor cells, it has been reported that their metastatic potential is related to actin skeleton arrangement and remodeling (17). In addition, to investigate cytoskeleton changes in the molecular level, we examined gene expression of *VIM* and *RHOA*. 

Vimentin is a cytoskeleton compound playing important role in the migration and invasion of breast cancer cells. Similar to the finding obtained in this experiment, an increased expression of *VIM* has been described as a epithelial to mesenchymal transition marker, leading to the enhanced invasion and metastasis in breast cancer cells. This may be one of the reasons why increased expression of *VIM* in breast cancer contributes to chemoresistance and poor prognosis (18). It is required to be furher elucidated whether *VIM* overexpression, under simulated microgravity, is indeed a marker of increased invasiveness. Thus metastatic potential is increased, or it rather reflects loss of surface attachment and spheroid formation without changes in metastatic ability. However, targeted and selective cytoskeleton derangement in cancer cells (e.g. by RNA-interference) could be a potential tool in future tumor therapy. As a therapeutic approach in this field, microRNA targeting against *VIM* was shown to decrease breast cancer invasion in animal studies (19). RhoA is another small GTPase with several functions, and it is known to be a key effector in the polymerization of actin filaments (20). Hence, we also examined *RhoA* gene and protein expressions. 

In addition to these changes related to the cytoskeleton, we observed further various alterations in gene expression and corresponding protein synthesis. Firstly, we normalized qRT-PCR data to 18s rRNA and western blot data to GAPDH expression. Although it is not exactly known which gene or protein undergoes the least changes under simulated microgravity, the indicated housekeeping genes look the best option in this experiment, due to several reasons: 18s rRNA is known to be very stably expressed under many different circumstances and it has been used as a housekeeping gene in simulated microgravity research by the other groups before (10). GAPDH has also been previously used for normalization of western blot data in microgravity research by the other groups (21). Indeed, the corresponding bands of GAPDH protein showed very similar intensity in our experiments. In the endeavor to obtain information whether the breast cancer cells transform towards a more or less malignant phenotype under simulated microgravity, we measured a variety of genes known as proto-oncogenes, tumor suppressor genes, or those which are related to cell proliferation and differentiation. *BRCA1*, with no mutation and with normal function, is a well-known gene counteracting genome instability and acting as a tumor suppressor gene (22). 

We observed significant overexpression of *BRCA1*, at least on the gene level, in the spheroids. This could be indicative of a transformation towards a phenotype with improved genomic repair and stability. Further experiments are required to evaluate this question in more detail. *BRCA1* gene expression was even higher in the spheroids than adherent cells, while this is not observed in the protein level. It is proposed that adherent cells under simulated microgravity might be a precursor of spheroids, turning into spheroids later. However, the adherent cells also could be an own entity for reasons unknown hitherto, remaining them adherent for a long period of time or even permanently. In a study, mouse embryonic stem cells (mESCs) were exposed to microgravity during spaceflight for 15 days. Analysis of this study showed down-regulation of *BRCA1* gene (23), Although it still need to evaluate whether BRCA1 mimics similar tasks in mESCs and the breast cancer cells we examined. On the other hand, we observed significant up-regulation of *ERBB2* gene, but not HER2 protein, particularly in the spheroids. The cell line we used overexpresses HER2 anyway from the beginning. HER2 proteins consist of trans-membrane growth factor receptors activating intracellular signaling pathways. HER2 has been shown to play an important role in the pathogenesis of human breast cancer, and overexpression of this protein in human breast cancer cells is usually related to more aggressive behavior. Measurement of *ERBB2*/HER2 expression therefore seems of great interest to us, as this could give a hint towards transformation into a more aggressive phenotype induced by simulated microgravity. 

As spheroid formation is associated with detachment of cells from a confluent 2D state, it is proposed that similar mechanisms to metastasis formation from a solid tumor come into action. We therefore measured *RAB27A* gene expression and Rab-27a protein production, as it is known to play a crucial role not only in breast gland development, but also in breast cancer pathology, particularly in modulation of metastatic potential (24). As a small GTPase, Rab-27a controls various steps of exosome release, and exosome-mediated intercellular communication plays a crucial role in the above described processes. In our experiments, we could not detect any significant alteration in this gene and protein. The reasons still have to be elucidated, but a different exposure time to simulated microgravity possibly could show expression changes in future studies. To our knowledge, we chose a negative estrogen receptor cell line in our experiments and exposed it to simulated microgravity for the first. MAPK1/MAPK1 therefore seem particularly interesting measurement targets, as MAPK1 is one of the effectors in breast cancer cell estrogen signaling (25).

We observed significant up-regulation of *MAPK1*/ MAPK1 on both gene and protein levels under simulated microgravity. It has been shown that inhibition of MAP kinase pathway can lead to conversion of negative estrogen receptor breast cancer cells, as with our experiments, to a positive estrogen receptor phenotype (26). Up-regulation of that could therefore be either interpreted in the sense of the negative estrogen receptor preservation, potentially more malignant phenotype, or the MAP kinase pathway take over further tasks in the microgravity setting which still needs to be elucidated. Despite not obtaining statistical significance, down-regulation of *VEGF* gene expression and protein production under simulated microgravity is another interesting finding. VEGF is known to be a potent endothelial growth factor regulating vascular permeability. Particularly, high VEGF expression is known to be associated with tumor progression and poor prognosis of breast neoplasms in the clinical setting (27). So that, *VEGF* gene and protein expression levels seem to be further important targets to evaluate. Anti-VEGF drugs (such as bevacizumab) are already administered in cancer therapy, and identification of the mechanisms responsible for *VEGF* down-regulation could help pave the way for new therapeutic strategies in this area.

Overall, we observed a variety of changes in gene and protein expressions. Most of these changes, particularly regarding the cytoskeleton, were indicative of a more invasive and aggressive phenotype. Up-regulation of *BRCA1*, as a tumor-suppressor gene in the non-mutated state, is contradictory to these findings to some extent, and significance of that needs to be further investigated. Utilizing western blot, a strong correlation was determined between alterations of gene expression and corresponding protein content in *VIM*/Vimentin, *RAB27A*/Rab-27a and *MAPK1*/MAPK1. BRCA1 and HER2 showed no detectable increase in protein content, despite up- regulation in gene expression level under microgravity. Surprisingly, there was decreased protein level of RhoA under simulated microgravity, despite up-regulation of gene expression. There are several possible explanations for this observation. One potential reason is the existence of several post-translational modifications. Different half- lives of proteins could be the other potential explanation. Rapid degradation of mRNA or delayed protein synthesis could also be conceived. RhoA protein, in particular, has been reported to undergo significant optional alteration in its half-life by post-translational methylation (28). Similar reasons my also account for the differences that we detected between western blot and qRT-PCR results for *BRCA1*/BRCA1 and *ERBB2*/HER2.

Interestingly, another study reported a general decrease of protein synthesis under microgravity for the yet unknown reasons (29). Simulated microgravity appears as an easy-to use and interesting 3D culture model for in vitro studies of breast cancer cells. Elongated shape of the spheroids resembles natural tumor structure better than conventional *in vitro* culture. Besides, both tumor- suppressor genes and proto-oncogenes were altered in their expressions. Better understanding of the underlying mechanisms could help develop new tools to selectively influence proliferation, differentiation and invasion of breast cancer cells and pave the way for new therapeutic options.

## Conclusion

Simulated microgravity induces spheroid formation in human breast cancer cells. Here we observed substantial changes in cytoskeleton morphology, cytoskeleton related gene and protein expression. We also determined change in gene and protein expression levels of proto-oncogenes and tumor suppressor genes. Our experiments could be a step towards a versatile, easy-to handle 3D culture model of human breast cancer. It could provide new insights in the molecular mechanisms of breast cancer pathogenesis paving the way to new therapeutic strategies. 
